# Migration of ingested sharp foreign body into the bronchus: a case report and review of the literature

**DOI:** 10.1186/s12890-021-01458-x

**Published:** 2021-03-17

**Authors:** Yuanhua Qiu, Shan Xu, Yafang Wang, Enguo Chen

**Affiliations:** Department of Respiratory and Critical Care Medicine, Regional Medical Center for National Institute of Respiratory Disease, Sir Run Run Shaw Hospital, School of Medicine, Zhejiang University, Hangzhou, Zhejiang Province China

**Keywords:** Esophageal foreign body, Rigid esophagoscope, Fiberoptic bronchoscope, Holmium laser

## Abstract

**Background:**

Foreign body ingestion is a common emergence in gastroenterology. Foreign bodies are most likely to be embedded in the esophagus. The sharp ones may penetrate the esophageal wall and lead to serious complications.

**Case presentation:**

A 72-year-old Chinese female was admitted to our hospital with a 4-day history of retrosternal pain and a growing cough after eating fish. Chest computed tomography scan indicated that a high-density foreign body (a fish bone) penetrated through the esophageal wall and inserted into the left main bronchus. First, we used a rigid esophagoscope to explore the esophagus under general anesthesia. However, the foreign body was invisible in the side of the esophagus. Then, the fiberoptic bronchoscopy was performed. We divided the fish bone, which traversed the left main bronchus, into two segments under holmium laser and removed the foreign body successfully. The operation time was short and there were no complications. The patient was discharged 1 week postoperatively and was symptom free even under a liquid diet.

**Conclusions:**

There are several challenges in the management of this rare condition. We applied the technique of interventional bronchoscopy to the management of esophageal foreign body flexibly in an emergency. A surgery was avoided, which was more invasive and costly.

## Background

Gastrointestinal foreign body incarceration as a common disease in emergency mostly occurs in pediatric department, with a high incidence in children aged 5 years and younger [1–3]. In adults, most cases of foreign body ingestion are associated with factors such as advanced age, mental disorder, mental retardation, alcohol consumption, or profit-seeking behavior by prisoners [1]. The occurrence of foreign body-related complications is closely associated with foreign body types, incarceration time and incarceration position [4]. Most foreign bodies (80–90%) can pass through the digestive tract by themselves without clinical intervention. About 10–20% of foreign bodies require endoscopic treatment. Less than 1% of patients require surgery [1, 5]. Since the first reported successful endoscopic removal of foreign bodies by McKechnie in 1972 [6], endoscopic treatment has become the first-choice treatment for foreign bodies in the upper digestive tract [7, 8]. Flexible endoscope and rigid endoscope are both widely used. Mouse forceps and snares are the most commonly used auxiliary devices to improve the success rate of treatment and reduce the incidence of operation-related complications. Few researches reported on the migration of esophageal foreign bodies into the bronchus. The joint management of foreign bodies by esophagoscope and bronchoscope faces a series of challenges. Here we reported a successful case. An elderly female patient who accidentally ingested a fish bone incarcerated in the esophagus and delayed medical treatment, leading to the fish bone puncturing through the esophageal wall and crossing the left main bronchus. Under general anesthesia, holmium laser assisted fiberoptic bronchoscope was used to successfully remove the fish bone when the expected effect was not achieved by rigid esophagoscopy. It is hoped that this study can attribute to the management of some special cases of esophageal foreign bodies in the future.

## Case presentation

**Fig. 1:**
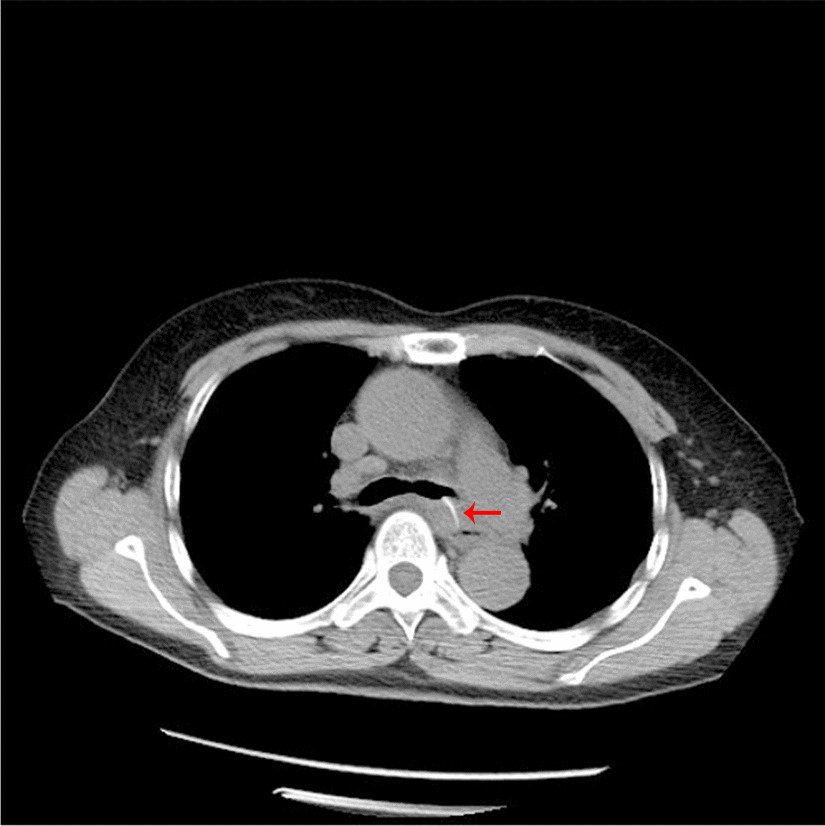
Plain axial CT scan of the chest. The chest CT image shows that a slender fish bone crosses the esophageal wall and considered involves the left main bronchial wall

**Fig. 2:**
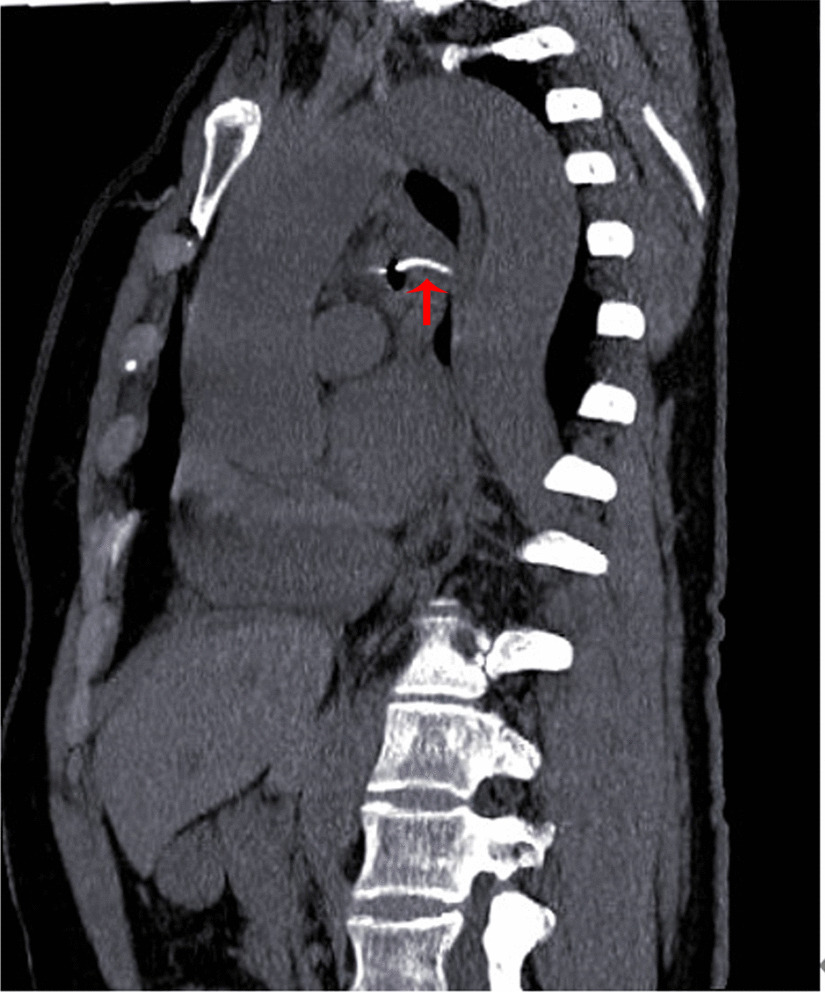
CT sagittal reconstruction image of the chest. The figure shows that a slender fish bone traverses the esophagus, mediastinum, and left main bronchus

**Fig. 3:**
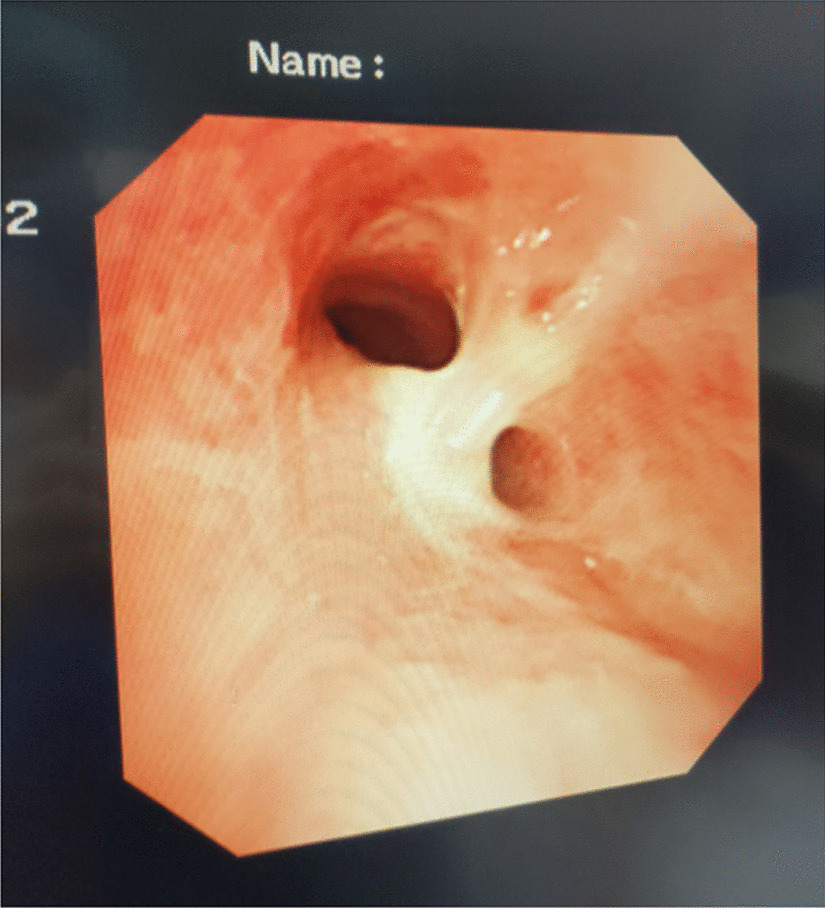
A foreign body in the proximal part and near the entrance of left main bronchus is seen crossing the bronchial wall during the operation under fiberoptic bronchoscope

**Fig. 4:**
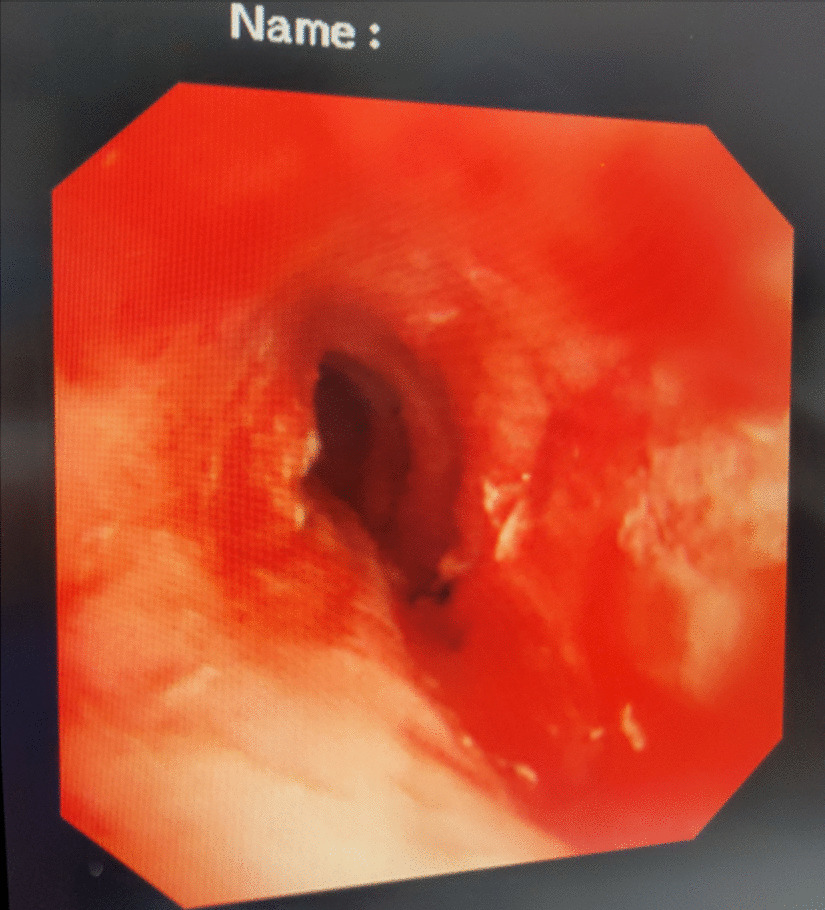
After removal of the foreign body, granulation tissue hyperplasia can be observed in local bronchial mucosal

**Fig. 5:**
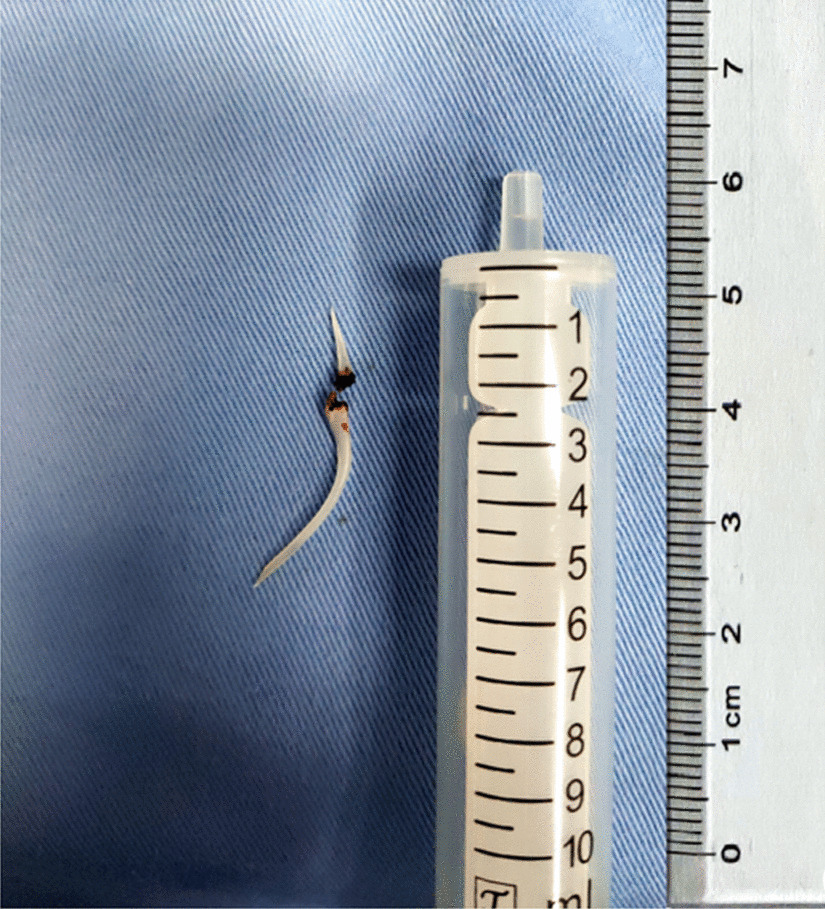
The foreign body is a sharp fish bone at a length of approximately 3 cm

A female patient, 72 years old, was admitted for “retrosternal pain for 4 days during eating” with a healthy history before. Four days ago, the patient had a foreign body sensation in the pharynx after eating freshwater fish. Then she ate as usual but felt retrosternal pain when eating. One day ago, the pain worsened after eating rice cake and was accompanied by severe irritant dry cough without fever, hemoptysis, or hematemesis. The patient was admitted to Regional medical center for National Institute of Respiratory Disease, Sir Run Run Shaw Hospital, School of Medicine, Zhejiang University. Chest plain computed tomography (CT) scan suggested “flat esophagus and bronchial bifurcation with a high-density shadow, a foreign body was considered with the length of 2.8 cm that crossed the left main bronchial wall, and the local bronchial wall mucosa thickened” (Figs. 1, 2). Treatment was supportive with fasting, fluid infusion, monitoring and so on. During the operation, a rigid esophagoscope (S121; Hangzhou Tonglu Medical Optical Instrument Co., Ltd, Zhejiang, China) was used under general anesthesia with endotracheal tube. It was observed that the mucosa ulcer was at the 2 o’clock direction in the esophagus 26 cm away from the incisors, with hyperemia and swelling of the surrounding mucosa. No obvious foreign body was found in the cavity. Under a fiberoptic bronchoscope (BF-260; Olympus Optical Co., Ltd, Tokyo, Japan) via the endotracheal tube, a needle-shaped foreign body (considering fish bone in combination with medical history) in the proximal part and near the entrance of left main bronchus could be seen intraoperatively, and penetrated the bronchial wall. Local bronchial wall showed granulation tissue hyperplasia, mucosal swelling and easy bleeding (Fig. 3). After the granulation tissue was cleaned intraoperatively and the fish bone was exposed, the holmium laser (VersaPulse 80/100 W PowerSuite; Lumenis Ltd, Israel) with the settings of 1 J and 8 Hz was used to broke the fish bone in the middle which was located in the left main bronchus, and foreign body forceps (JHY-FG-18-120-A4; Jiuhong Medical Instrument Co., Ltd. Changzhou, China) were used to remove the two segments, respectively. There was no significant active hemorrhage in the bronchus after operation (Fig. 4). The entire operation under the fiberoptic bronchoscope lasted for 20 min. The foreign body was a sharp and hard fish bone about 3 cm in length (Fig. 5). A jejunal nutrition tube was indwelling during the operation, and nasal-feeding nutrition as well as gastrointestinal decompression were given postoperatively. Secondary infection and leakage were also monitored. No obvious leakage was noted in upper gastrointestinal contrast half a month after the operation. Then, the jejunal nutrition tube was removed, and there was no obvious discomfort when having semi-fluid food through the mouth.

## Discussion

Due to the differences in diet culture, the proportion of fish bones in ingested gastrointestinal foreign bodies in some Asian countries such as China is significantly higher than that in Western countries [9–11]. Among Chinese populations, if fish bones are stuck in the digestive tract, treatments such as swallowing whole solid food will be used rather than emergency medical treatment, as the case in this study. Later, symptoms of the patient such as retrosternal pain and difficulty in eating were significantly aggravated, and a severe irritable cough appeared. It was probably the folk way that sent the fish bone deeper into the left main bronchus.

Sharp foreign bodies such as fish bones are usually incarcerated in the esophagus [10], especially in the upper esophagus [12, 13]. The outcomes are related to the three physiological strictures of the esophagus including the upper esophageal sphincter, the aortic arch or the left main stem bronchus, and the lower esophageal sphincter. Another study discovered that fish bones tended to be affected horizontally in the pharynx and esophagus, rather than in parallel or oblique positions, making them easier to penetrate the esophageal wall [11]. In this case, the ingested fish bone was linear in shape with two tapered ends, which was easy to penetrate the esophageal wall. The fish bone gradually moved to the deeper viscera over time, leading to greater harm.

Complications of esophageal foreign bodies mainly consist of esophageal mucosal injury, hemorrhage, infection and perforation, etc., which vary according to the involvement of surrounding organs and tissue. The incidence of the ingested sharp foreign bodies can be as high as 35% [1]. The most serious situation was that foreign bodies penetrate into the esophageal wall and the aorta, leading to the aortoesophageal fistula (AEF), which will cause death by the massive hemorrhage [13]. Our case was that the ingested fish bone stayed close to the second stricture of the esophagus, penetrating the wall and mediastinum, involving the left main bronchus, and contributing to the esophagobronchial fistula (EBF). Because of the small fistula, only a period of postoperative fasting was required without further treatment.

The method to remove gastrointestinal foreign bodies is associated with the type and size of foreign bodies, position and time of incarceration, the state of patients and the habits of doctors [12, 13]. Flexible endoscopy is the first choice for examination and treatment of upper gastrointestinal foreign bodies, with a high success rate (greater than 94%) and fewer complications (0–6%) [1, 10]. While rigid endoscopy is more suitable to deal with sharp and long foreign bodies. The rigid endoscopy has some advantages over the upper esophageal sphincter or hypopharyngeal foreign body incarceration, while the lower esophageal foreign bodies should be treated by flexible endoscopy [14]. A recent meta-analysis compares the treatment of foreign bodies in the upper esophagus by flexible and rigid endoscopes, and the success rates and overall complication rates of both are similar [15]. The habits of doctors are also quite different [13]. In adults, rigid endoscopy are often used as a second-line treatment after failure of flexible endoscopy, or as a first-line treatment in more difficult situations, but 85.7% of patients in a Greek study received rigid endoscopy treatment [13]. In this case, the fish bone penetrated the esophageal wall, posing certain challenges to the operation of rigid esophagoscope.

For sharp foreign bodies like fish bones, emergency grading of endoscopic intervention time (preferably within 2 h, but at latest within 6 h) is required [1, 16]. It is generally recommended to avoid endoscopic operation and adopt surgical treatment if endoscopic treatment involves perforation, high risk of bleeding or foreign bodies having been deeply inserted into the esophageal wall [17]. In the case of esophageal foreign bodies, the proportion of fish bone involved trachea and bronchus is relatively low without clear literature reports, and the guidelines have not proposed specific operation methods for this issue, which poses a greater challenge to the diagnosis and treatment of this case. With the support of general anesthesia, rigid esophagoscopy was firstly used to explore the esophagus by the otorhinolaryngologist. During the operation, the foreign body was completely invisible at the esophageal end, and only local mucosal rupture and swelling was observed, indicating that the fish bone had been deeply inserted into the esophageal wall. According to the conclusion of previous multi-disciplinary discussions, we then decided that fiberoptic bronchoscope should be used to remove the foreign body by a respiratory physician.

Rigid bronchoscopy would have been the ideal method for removal of a sharp foreign body in the airway [18]. However, in this case, we did not choose it since endotracheal intubation and rigid esophagoscope were applied. Under fiberoptic bronchoscope, the fish bone had crossed the left main bronchus, with both pointed ends punctured into the bronchial wall. We had tried to regress the bone back into the esophagus, but it was difficult to expose the bone to the esophagus side. Advances in bronchoscope, such as laser technology, have broadened the therapeutic options. For adults, laser therapy is mainly applied in the treatment of airway tumors, bronchial stones and so on [19]. In the pediatric field, its most common indication is granuloma clearance before tracheal fistula removal [20]. As for treatment of foreign bodies with lasers, no relevant guidelines or recommendations have been found. Only some case reports in pediatrics can be consulted. Chicken bone in a 19-month-old girl and pistachio shell in a 7-year-old boy were successfully removed by bronchoscope with the assistance of neodymium (YAG LASER), respectively [21, 22]. In this case, we found both ends of the fish bone had been deeply penetrated left main bronchus under fiberoptic bronchoscope. Bronchial mucosa hemorrhage was obvious with forced removal of the fish bone. Therefore, we used the holmium laser to break the foreign body, thus removing the complete fish bone, successfully avoiding surgery, and reducing complications as well as medical costs.

## Conclusion

This study reported a case of esophageal foreign body invading the left main bronchus, which was successfully removed with the help of holmium laser via fiberoptic bronchoscope, hoping to provide reference for some special cases of esophageal foreign bodies. When ingested foreign bodies involve trachea or bronchus, it is a feasible, efficient and minimally invasive method to remove foreign bodies with assistance of bronchoscope. The application of holmium laser can effectively break the hard foreign bodies while hold them in a fixed position relatively, making the operation more convenient, shortening the operation time and reducing the occurrence of complications such as hemorrhage. Additionally, multi-disciplinary cooperation plays a key role in the ideal outcome.

## Data Availability

The data used to support the findings of this study are included within the article. The data and materials in the current study are available from the corresponding author on reasonable request.

## Abbreviations

CT: Computed tomography
YAG: Yttrium aluminium garnet
LASER: Light amplification by stimulated emission of radiation
